# Percutaneous Radiofrequency Ablation Combined With Chemotherapy Versus Chemotherapy Only for Ovarian Cancer Liver Metastasis

**DOI:** 10.3389/fonc.2021.793024

**Published:** 2022-01-03

**Authors:** Chun-Xue Wu, Miao-Ling Chen, Hao Zhang, Jian-Jun Han

**Affiliations:** ^1^ Shandong First Medical University and Shandong Academy of Medical Sciences, Shandong, China; ^2^ Interventional Radiology Department, Shandong Cancer Hospital and Institute, Shandong First Medical University and Shandong Academy of Medical Sciences, Shandong, China

**Keywords:** radiofrequency ablation, ovarian cancer liver metastasis, chemotherapy, combined therapy, survival analysis

## Abstract

**Purpose:**

To compare the feasibility and efficacy of radiofrequency ablation (RFA) combined with chemotherapy and chemotherapy alone in patients with ovarian cancer liver metastasis (OCLM).

**Methods:**

In this retrospective study, a total of 60 patients diagnosed with OCLM between May 2015 to February 2017 were included. All patients with ovarian cancer received chemotherapy and primary cytoreductive surgery before. Thirty patients underwent RFA and chemotherapy, and thirty patients only took chemotherapy. The overall survival (OS), CA-125 levels, and serum AST and ALT levels were compared between the two groups.

**Results:**

In the RFA group, the 1-,2-, and 3-year OS rates after RFA were 93.3%, 80.0%, and 53.3%, respectively. Serum AST and ALT levels were both elevated after RFA (p=0.0004, p<0.0001). In the chemotherapy group, the 1-,2-, and 3-year OS rates were 79.5%, 60.1%, and 42.1%, respectively. Levels of serum AST and ALT were stable. CA-125 levels for both groups were also available.

**Conclusion:**

Based on our analysis of a single institution’s series of patients with OCLM, RFA could be a feasibly effective option in the management of OCLM.

## Introduction

Ovarian cancer (OC) is the seventh most common cancer and the eighth leading cause of cancer-related deaths in women worldwide (1). OC has historically been called the “silent killer” because it often occurs with no clinical symptoms until it had progressed to advanced stages (2). Although cytoreductive surgery and adjuvant therapy are increasing median progression-free survival and overall survival (OS), the OS has been affected only modestly. Most patients still died of disease progression and metastasis (3). A recently published review shows that 5-year OS rates of stage IV ovarian cancer was about 28% (4). OC metastasis through the intraperitoneal route of dissemination has been recognized as the most common pattern of extraovarian tumor spread, and it may also metastasize to distant organs, such as liver, lung, bone, and brain, through hematogenous seeding at the time of diagnosis or during the follow-up (5). In addition, the liver is one of the most common metastatic sites for advanced OC (6). It is reported that patients with ovarian cancer liver metastasis (OCLM) underwent hepatic resection (HR) within secondary cytoreductive surgery had 3-year post-HR overall survival of 72.9% (7). Meanwhile, when hepatoceliac lymph node are involved surgical resection can also improve the survival time (8). Unfortunately, multiple liver metastases (LMs) with a depth of more than 1 cm were commonly evaluated as unresectable status (9). And the unresectable liver metastases conveys a very poor prognosis with a median survival within 1 years due to rapid tumor progression (10). Therefore, it is vital for these patients to choose the optimal treatment for liver metastasis.

As a promising thermal ablation modality, radiofrequency ablation (RFA) is used for more and more patients with hepatocellular carcinoma (HCC) and metastatic liver carcinoma as an effective local therapy, particularly when the patient’s liver functional reserve does not allow a radical resection (11–13). However, whether RFA can be used to treat OCLM needs to be explored. A study is needed to determine whether RFA can be effective in managing OCLM.

In this study, we compared the feasibility and effectiveness between RFA combined chemotherapy and chemotherapy only in patients with OCLM.

## Materials and Methods

This retrospective study was performed according to the principles of the Declaration of Helsinki. The protocol was approved by the ethics committee of Shandong Cancer Hospital (Shandong, China) (NO. SDTHEC20210207). Because of the retrospective nature of this study, patient consent for inclusion was waived.

### Patients

There are 30 patients with OCLM who underwent computed tomography (CT)-guided percutaneous radiofrequency ablation from May 2015 to February 2017, defined as the RFA group. The control group included 30 patients with OCLM who underwent chemotherapy in the same period. To increase the comparability of the control group and the RFA group., the inclusion criteria were set as follows: (1) up to 5 liver metastases and no one greater than 5 cm in diameter; (2) an Eastern Cooperative Oncology Group performance (ECOG) status score of 0 or 1; (3) Child-Pugh class A or B; (4) underwent previous cytoreductive surgery (CRS) and chemotherapy; (5) patients either ineligible or refused for liver surgical resection. The exclusion criteria were as follows: (1) underwent any local treatment for liver metastasis (2) progressive liver disease or liver failure. The liver metastasis was diagnosed by biopsy or clinical data (the OC history along with the new lesion in the liver with typical imaging findings, that is, peritumoral enhancement in arterial-phase and hypoenhancing on dynamic). The histologic types of primary lesions were also reviewed. In the RFA group, 6 patients were serous cell type, 22 patients were mucinous cell type, and 2 patients were endometrioid cell type. In the control group, 7 patients were serous cell type, 20 patients were mucinous cell type, and 3 patients were endometrioid cell type.

The following information was recorded from patients: age, ECOG score, FIGO stage, histologic type, number of tumors, serum CA-125 level, Child-Pugh stage, platinum-free interval, site of metastasis, and laboratory examination (serum alanine transaminase [ALT] and aspartate transaminase [AST] levels) related to hepatic function. Serum CA-125, AST and ALT levels was recorded before and after chemotherapy and RFA treatment. All the information came from the medical records database of Shandong cancer hospital.

### RFA Procedure

For the RFA group, all patients received RFA therapy as local therapy and chemotherapy based on platinum as systemic therapy (**Table 1**).

**Table 1:** Chemotherapy treatment for liver metastasis in the two groups.

Chemotherapy	RFA group	Chemo group
**Paclitaxel 175mg/m^2^ + carboplatin AUC 5~6**	7	9
**Caelyx 30 mg/m^2^+ carboplatin AUC 5~6**	12	11
**Docetaxel 60~75mg/m^2^+ carboplatin AUC 5~6**	11	10

A 128-slice spiral CT scanner was utilized to guide RFA puncture and acquire images. A Cool-tip™ RF ablation system (Medtronic, USA) including one single 17-gauge electrode, two grounding pads, and inflow and outflow tubing set was used.

RFA was performed under local anesthesia with 5 mL of 1% lidocaine plus conscious analgesia sedation, which was induced by intravenous administration of 0.1 mg of fentanyl (Yi Chang Pharmaceutical, Hubei, China). The grounding pad was stuck to each thigh of the patient. Using CT guidance, the radiofrequency electrode was carefully introduced into the target lesion at the predetermined location. The number of electrodes was dependent on tumor size, shape, and location. The purpose of treatment was to make the low-density area produced by RFA cover the treated tumor larger than 0.5 to 1 cm. After the ablation of the target tumor, the needle track was carefully treated, which needed to continue ablation while pulling out the needle to prevent bleeding and tumor spread through the needle track.

Patients were hospitalized for 1 to 2 days after RFA unless their complications necessitated longer hospitalization. A CT scan was performed the next morning after treatment, which might allow an initial evaluation of the ablated zone to determine whether there was any remaining malignant tissue that would require an additional ablation and whether there has bleeding or other complications needed to be taken care of.

### Chemotherapy Procedure

All patients from the two groups received intravenous platinum-based chemotherapy which was repeated every three weeks for about six cycles (**Table 1**). Individual-specific treatment plans depended on their tumor stage, previous treatment, and physical endurance. In RFA group, the average chemo cycles were 3.8 before RFA and 6.7 during the treatment, while the control group had an average of 7.2 cycles during the treatment.

### Statistical Analysis

Data analysis was performed in GraphPad Prism 8.0.2. Student’s t-test was used to compare the data between the RFA and chemo groups. The Kaplan–Meier method with the log-rank test was utilized to evaluate OS. *p*<0.05 was considered statistically significant.

## Results

### Patients and Tumor Response

**Figure 1:**
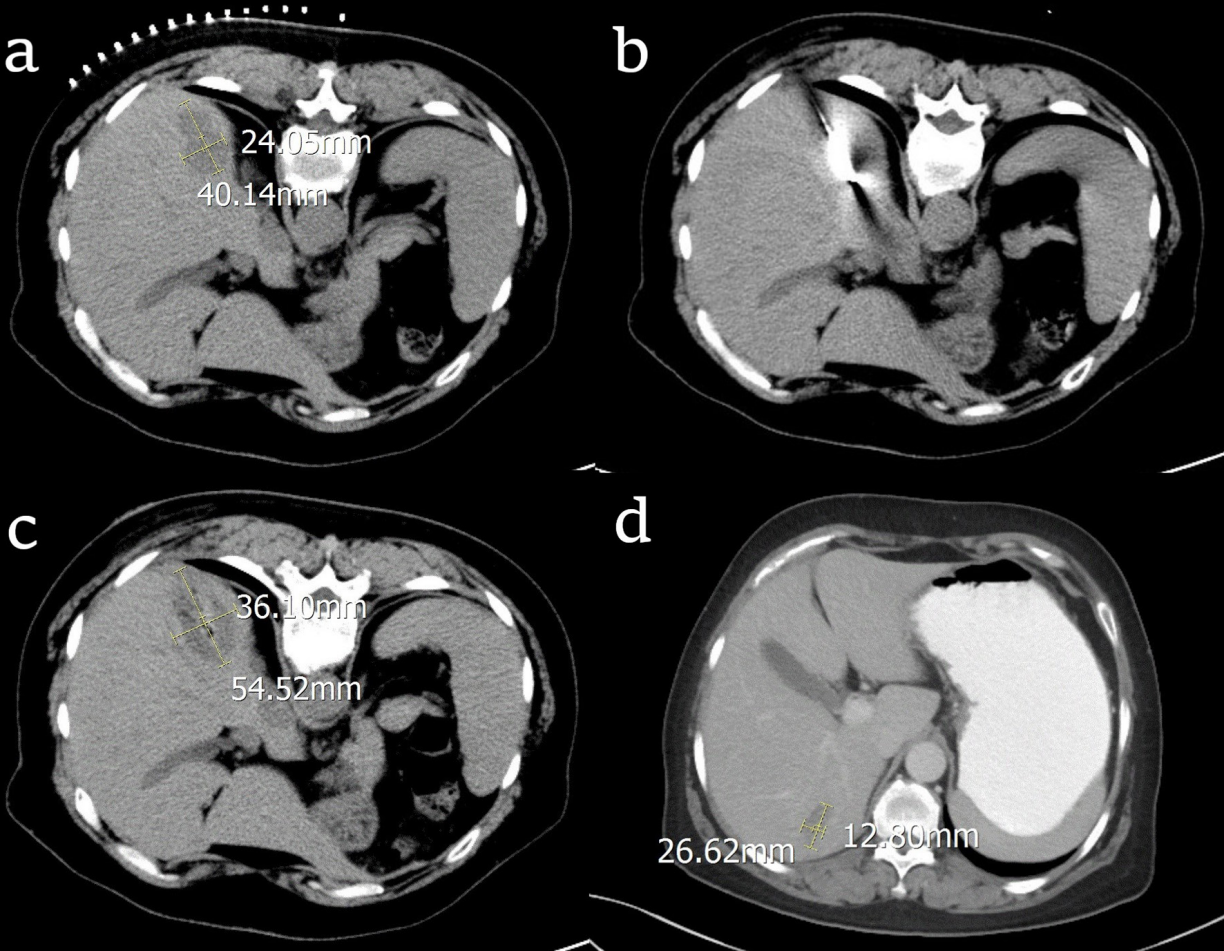
CT images of the process of radiofrequency ablation (RFA) treatment for one patient with ovarian cancer liver metastasis (OCLM). **(A)** the location and diameter of the tumor before RFA; **(B)** the radiofrequency electrode in the center of the tumor; **(C)** the location and diameter of the tumor right after RFA; **(D)** the location and diameter of the tumor after 6 months.

Baseline patient characteristics were generally well balanced between the two treatment groups (**Table 2**). There were fifty liver metastases nodules that were ablated in RFA group. In the RFA group, all the patients received the optimal RFA treatment, which was provided by multiple disciplinary teams by synthesizing the patients’ tumor burden, physical and financial condition. Nine patients had liver metastasis at the time of initial diagnosis of OC, 5 of them underwent neo-adjuvant chemotherapy before primary surgery, 4 of them received chemotherapy after primary surgery. Among the 9 patients, 3 of them had liver metastases only, 2 patients had pelvic metastasis, and 3 patients had abdominal metastases. The other 21 patients found liver metastasis at the first diagnosis of recurrence of OC, 8 patients had pelvic metastasis, 13 patients had abdominal metastases, 6 patients underwent second CRS before RFA treatment for the liver metastasis. All 21 patients were treated with RFA. The median platinum-free interval of them was 20 months (range 8-35 months). During the follow-up period, 12 patients had new metastases at other parts of the liver and 13 patients had systemic metastases. The process of RFA treatment for one patient was shown in **Figure 1**. In this case, the radiofrequency electrode was positioned to pass through the tumor’s center along its largest diameter, providing an ablation area that extends 0.5-1 cm beyond the tumor. The tumor was 40.14mm×24.05mm before RFA. After treatment, the RFA area was 54.52mm×36.10mm, which means the RFA treatment was complete. After 6 months, the necrosis caused by RFA was gradually absorbed and the volume was reduced to 26.62mm×12.30mm.

**Table 2:** Characteristics of patients.

Characteristics	RFA group	Chemo group
**Number of patients**	30	30
**Mean age, Year**	55 (range 38-74)	57 (range40-76)
**ECOG score**		
** 0**	28	26
** 1**	2	4
**FIGO Stage**		
** III**	17	19
** IV**	13	11
**Cell type**		
** Serous**	6	7
** Mucinous**	22	20
** Endometrioid**	2	3
**Number of Tumors**		
** Solitary**	14	12
** Multiple**	16	18
**Previous CA-125 level (U/mL)**		
** Average before CRS**	849.8	812.0
** after CRS**	19.63	21.30
**Child-Pugh**		
** A**	28	26
** B**	2	4
**Distribution of disease**		
** Liver only**	3	1
** Liver + pelvis**	10	15
**Liver + abdomen**	17	14
**Median platinum-free interval (month)**	20 (range8-35)	19 (range 6-31)

RFA, radiofrequency ablation; Chemo, chemotherapy; ECOG, Eastern Cooperative Oncology Group performance; FIGO, Federation International of Gynecology and Obstetrics; CRS, cytoreductive surgery.

**Figure 2:**
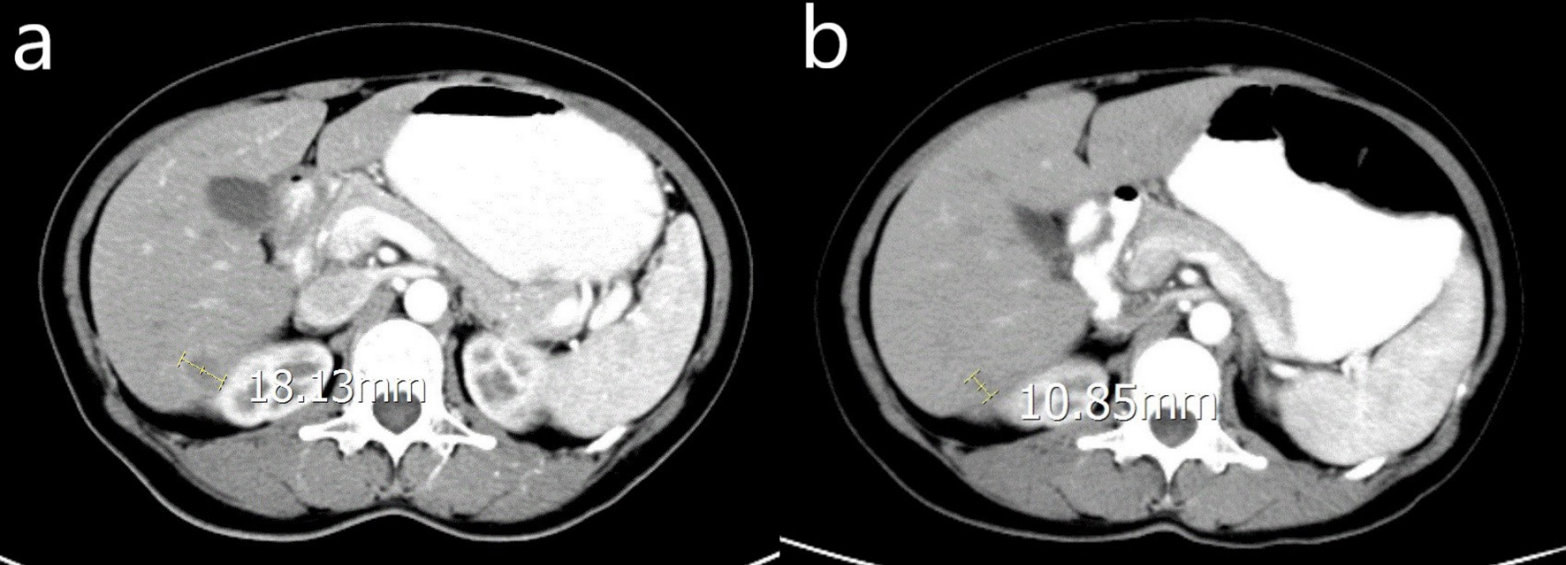
CT image of a woman with OCLM underwent chemotherapy only. **(A)** the location and diameter of the tumor before chemotherapy; **(B)** the location and diameter of the tumor one month after chemotherapy.

In the chemo group, 5 patients had liver metastasis at the time of initial diagnosis of OC, one of them had liver metastases, 2 of them had pelvic metastases, and 3 of them had abdominal metastases. After CRS and chemotherapy, the liver metastases were reduced. Twenty-five patients had liver metastases at first recurrence, 14 of them had pelvic metastases combined with liver metastases, 11 patients had abdominal metastases including liver metastases, 4 patients received the second CRS. The liver lesions were shrinking in 16 patients and stable in the others. The median platinum-free interval of them was 19 months (range 6-31months). During the follow-up period, the original liver metastases nodules progressed in 19 patients and new liver metastases occurred in 10 patients. 15 patients had systemic metastases including lung, bone, and brain. The CT images in **Figure 2**, showed a patient who took chemotherapy only, and the tumor was reduced from 18.13mm to 10.85mm.

### Value Distribution of Serum CA-125 in Both Groups

**Figure 3:**
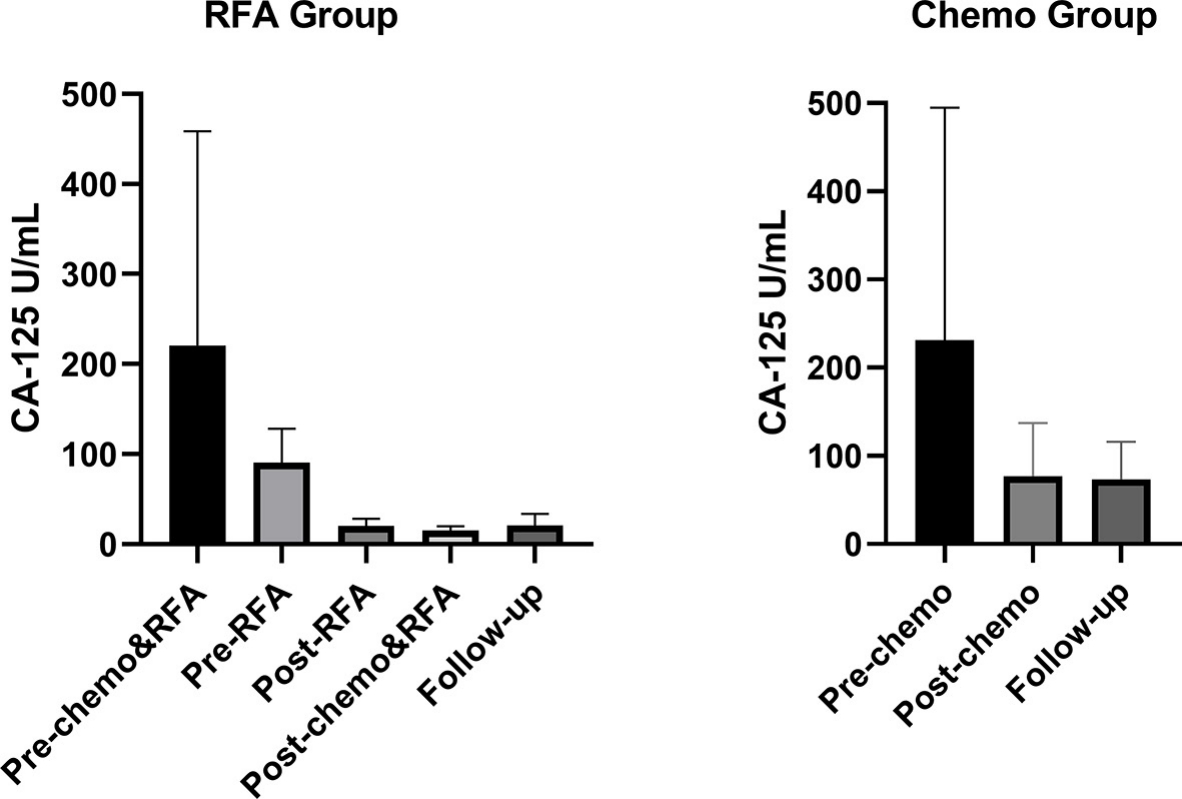
Serum CA-125 levels for all patients during the treatment. For the RFA group, pre-chemo&RFA shows CA-125 levels before chemo and RFA treatment, pre-RFA shows CA-125 levels before RFA, post-RFA shows CA-125 levels after RFA and before the next cycle of chemo therapy, post-chemo&RFA shows CA-125 levels 30 days after chemo and RFA. For the control group, pre-chemo shows CA-125 levels before chemo therapy, post-chemo shows CA-125 levels 30days after chemo therapy.

Results of CA-125 for both groups were shown in **Figure 3** and **Table 3**. For the RFA group, serum CA-125 levels were available before chemo and RFA (pre-chemo&RFA), before RFA (pre-RFA), after RFA and before the next cycle of chemo(post-RFA), and 30 days after chemo and RFA (post-chemo&RFA). CA-125 level was drop from 220.5 ± 238.0 to 15.2 ± 4.916 after chemotherapy and RFA, and from 90.70 ± 37.25 to 20.15 ± 7.992 after RFA. For the chemo group, serum CA-125 levels were available before chemo (pre-chemo) and 30 days after chemo (post-chemo) decreasing from 231.1 ± 263.8 to 76.91 ± 60.19. And for both groups, the follow-up CA-125 levels were available in 3-6 months, if the patient has more than one record we took the average value. Before chemotherapy and RFA the CA-125 levels in both groups showed no statistical difference(*p*=0.8707), while after chemotherapy and RFA and in the follow-up showed significant difference(*p*<0.0001, *p*<0.0001).

**Table 3:** serum CA-125 levels (U/mL) for all patients.

	Pre-chemo&RFA	Pre-RFA	Post-RFA	Post-chemo&RFA	Follow-up
**RFA group**	220.5 ± 238.0	90.70 ± 37.25	20.15 ± 7.992	15.2 ± 4.916	20.62 ± 13.03
**Chemo group**	231.1 ± 263.8	–	–	76.91 ± 60.19	73.64 ± 42.46
***p*-value**	0.8707			0.0001	0.0001

### Changes in Hepatic Function

As hepatic function markers, serum AST and ALT levels were assessed before chemotherapy and RFA (pre-chemo&RFA) and after chemotherapy and RFA (post-chemo&RFA) post-treatment in all patients showed in **Table 4**. There were no statistical differences on AST and ALT levels between the RFA group and chemo group before treatment (*p*=0.7299, *p*=0.7498). After treatment, the AST and ALT levels showed a significant difference (*p*<0.0001, *p*<0.0001).

**Table 4:** Serum AST and ALT levels for all patients.

Group	AST mean ± SD (U/L)		*p*-value	ALT mean values ± SD (U/L)		*p*-value
	Pre-chemo&RFA	Post-chemo&RFA		Pre-chemo&RFA	Post-chemo&RFA	
**RFA group**	43.84 ± 18.54	63.72 ± 22.53	0.0004	46.94 ± 23.42	73.72 ± 30.04	0.0001
**Chemo group**	42.07 ± 21.05	41.14 ± 17.45	0.8508	45.13 ± 20.13	45.33 ± 21.73	0.9716
***p*-value**	0.7299	0.0001		0.7498	0.0001	

RFA, radiofrequency ablation; Chemo, chemotherapy; AST, alanine transaminase; ALT, aspartate transaminase.

In the RFA group, the AST and ALT levels were sharply promoted after RFA and chemotherapy and have a significant statistical difference compared to before RFA and chemotherapy (*p*=0.0004, *p*<0.0001). These outcomes indicate that RFA would do some harm to liver function making AST and ALT levels increase.

In the chemo group, AST level and ALT level have no statistical difference (*p*<0.8508, *p*<0.9716) before and after treatment.

### Survival

**Figure 4:**
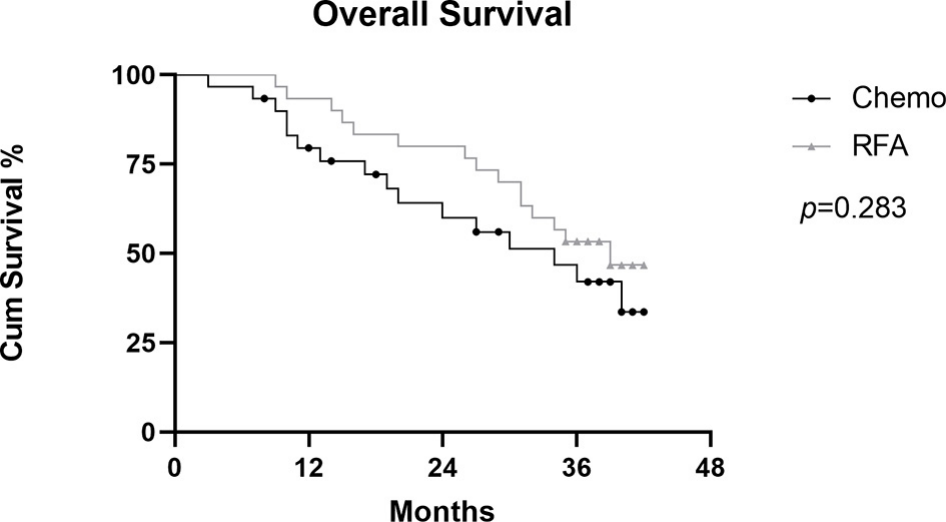
Graph shows comparation of cumulative Overall survival (OS) between RFA group and chemo group in patients with OCLM.

**Figure 5:**
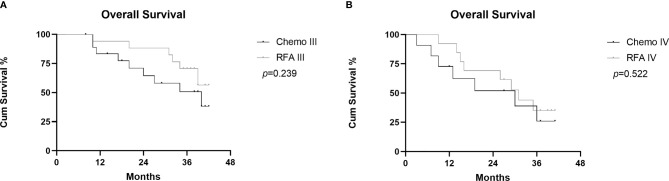
Graphs show subgroup analysis of cumulative OS between RFA group and chemo group in FIGO III stage patients **(A)** and FIGO IV stage patients **(B)**.

In the RFA group, the 1-,2-, and 3-year OS after RFA were 93.3%, 80.0%, and 53.3%, respectively. During the follow-up, 15 patients died because of disease progression. In the chemo group, the 1-,2-, and 3-year OS rates were 79.5%, 60.1%, and 42.1%. During the follow-up, 16 patients died because of disease progression (**Figure 4**). There was no statistical difference shown between the two groups(p=0.283). Median OS was longer in the RFA group than in the chemo group (34.0 and 39.0, HR=0.8718, 95%CI: 0.4310-1.763). **Figure 5A** shows OS of FIGO III stage patients from RFA group and chemo group (*p*=0.239), and **Figure 5B** shows the OS of FIGO IV stage patients from the two groups (*p*=0.522). Although the OS of RFA group was improved compared with chemo group, it was not enough to show the statistical difference.

## Discussion

To our knowledge, this is the largest series focused on the role of RFA in the management of OCLM. Another highlight of this study is that the control group was set to compare the efficacy of RFA with chemotherapy and chemotherapy alone for OCLM. Moreover, the present study demonstrates that RFA provides a viable effective option for patients with liver metastasis from OC.

In this study, we expanded the sample size and set the chemotherapy group as a control group. We examined the OS and the changes of tumor marker for 30 patients who received RFA and chemotherapy and 30 patients who received chemotherapy only. RFA group showed a more favorable OS rate with the 1-,2- and 3-year OS rates of 93.3%, 80.0%, and 53.3%, compared with chemo group of 79.5%, 60.1%, and 42.1%. Median OS was longer in the RFA group than in the chemo group (34.0 and 39.0, HR=0.8718, 95%CI: 0.4310-1.763). Although there was no statistical difference shown between the two groups(*p*=0.283), the extension of median survival showed clinical benefits. The median OS in the RFA group is slightly longer than that reported after liver resection in another study (media OS: 38.0 months) (14) Although the survival rate has improved, the reason why there is no significant difference in statistics may be that the data was based on a single center and the sample size is insufficient, and RFA only controls liver metastases, and metastases in other parts may still occur.

Besides, the tumor marker, CA-125, was decreased further after treatment in the RFA group. Based on these, we can conclude that RFA can not only improve overall survival but also reduce the level of serum CA125. CA-125 has been well established as an accurate and reliable method for monitoring patient response to treatment and confirming relapse in ovarian cancer patients (15). RFA treatment for liver metastases resulted in a significant decrease in serum CA-125 level. It is probably because that RFA can not only effectively kill tumor cells but also release tumor antigens that can provoke a systemic immune response. RFA can induce massive necrotic cell death which might activate immunity and the presentation of cryptic antigens to induce tumor-specific T cell response. RFA induces inflammatory effects that prevent the cycle of immune evasion by creating a substantial *in situ* source of acute inflammatory signals and tumor antigens in the form of necrotic tumor cells and cellular debris that generate systemic immunity (16). The immune response provoked by the localized RFA treatment maybe can cause a therapeutic effect on distant primary lesions. This may be the reason why CA-125 decreased further after RFA. This distant effect was mentioned in another report on liver metastasis of colorectal cancer (17). Furthermore, RFA was reported to markedly increase the infiltration of intratumoral CD8^+^T lymphocytes and the number of antigen-specific CD8^+^T cells within the tumor microenvironment, also it could increase CD8^+^ effector T cell infiltration at residual tumor sites (18–20).

However, RFA would simultaneously kill a large number of normal liver cells while killing the tumor cells. To accomplish the goal of complete ablation, the RFA area should be larger than the tumor margin, which would temporarily compromise liver function. In this study, despite similar initial liver function, RFA caused greater disruption, as shown by the increased AST and ALT levels after RFA. Fortunately, the liver has a unique potential to fully recover from temporary liver injury. Abnormally elevated AST and ALT levels would return to the reference range within a certain period.

Liver metastasis of ovarian cancer is a challenging problem and associated with poor prognosis (21). Previous studies had demonstrated that liver surgical resection was an effective and safe treatment to provide a better long-term prognosis including prolonged OS and local tumor control (22–24). However, many patients are ineligible for surgery because of multiple liver lesions, bilobar distribution of liver metastases, or the presence of widespread extrahepatic disease. It was reported that optimal liver resection was achievable in only 16% of patients, and palliative resection showed no significant survival benefit (22, 25). On the other hand, chemotherapy, including intra-arterial continuous infusion, is the most commonly used therapeutic strategy for patients with advanced and recurrent OC. Cisplatin or carboplatin in combination with paclitaxel has emerged as a standard first-line treatment for advanced OC. The 24 months estimated probability of survival rate of patients under went this combination therapy every 3 weeks was reported to be 78.9% (26). Besides, conventional chemotherapeutic regimens often accompany severe side effects and fail in metastatic cancer treatment due to chemoresistance, as cancer cells eventually develop resistance to chemotherapeutic drugs (27). Therefore, further exploration of the optimal treatment strategy is necessary for the management of these cases.

In recent years, RFA has been considered a good alternative treatment for patients with unresectable liver metastases, especially from colorectal cancer (28) and pancreatic adenocarcinoma (29). RFA significantly prolonged survival time and improved the quality of life for patients. Then some scholars have applied RFA on the treatment of liver metastasis of ovarian cancer. Liu et al (30) reported a group of 11 patients with 22 liver metastases from OC treated with ultrasound-guided RFA. Gervais et al. (31) showed a series of 6 patients with OCLM accepted RFA. Yuan et al. (32) reported a cohort of 42 women with metastatic ovarian and non-ovarian gynecologic tumors including OCLM treated with thermal ablation. The main limitations of these studies are the lack of a control group that can assess the survival benefit of RFA and the small sample size. Although RFA has been confirmed as a viable treatment for OCLM, the lack of a control group to assess its survival benefit and the small sample size limit these studies. This situation prompted us to do the present study, tryed to increase the sample size, and set a control group. Results of our study confirm that RFA can be a promising treatment for OCLM, which can extend patient survival times.

The main limitation of this study is that it is a single-center retrospective study. Since this study used only one center, had a small sample size, and was retrospective, the statistical power of the comparison may have been reduced, so some associations may not have been detected. A larger multi-center prospective study is needed to confirm our results in the future. With the progress of immunotherapy and the immune effect of RFA, the combination therapy of RFA and immunotherapy for metastatic ovarian cancer is likely to be a research focus in the future; the survival time may be extended again.

In conclusion, RFA can provide better long-term efficacy than chemotherapy alone, the present results suggest and encourage that RFA could be a feasible and effective option in the management of ovarian cancer liver metastasis.

## Data Availability Statement

The raw data supporting the conclusions of this article will be made available by the authors, without undue reservation.

## Ethics Statement

The studies involving human participants were reviewed and approved by ethics committee of Shandong Cancer Hospital (Shandong, China). Written informed consent for participation was not required for this study in accordance with the national legislation and the institutional requirements.

## Author Contributions

J-JH gave topic selection and guidance, C-XW reviewed cases, made statistical analysis and wrote manuscripts, M-LC reviewed cases, and HZ gave clinical consultants. All authors contributed to the article and approved the submitted version.

## Funding

This study was funded by the National key R & D Program “International innovation cooperation among governments” special project (grant number 2018YFE0126500) and the Plan for Science and Technology Development of Ji'nan (grant number 201907124).

## Conflict of Interest

The authors declare that the research was conducted in the absence of any commercial or financial relationships that could be construed as a potential conflict of interest.

## Publisher’s Note

All claims expressed in this article are solely those of the authors and do not necessarily represent those of their affiliated organizations, or those of the publisher, the editors and the reviewers. Any product that may be evaluated in this article, or claim that may be made by its manufacturer, is not guaranteed or endorsed by the publisher.
